# Prediction of heavy metal biosorption mechanism through studying isotherm kinetic equations

**DOI:** 10.1038/s41598-023-28655-4

**Published:** 2023-01-28

**Authors:** Mostafa G. Fadl

**Affiliations:** Nuclear Materials Authority, El Maadi, P.O. Box 530, Cairo, Egypt

**Keywords:** Biotechnology, Microbiology, Biogeochemistry

## Abstract

The kinetic constants for free and immobilized cells were determined by measuring reaction rates at different metal concentrations at the optimum reaction conditions. (K_max_ and V_max_) were calculated from the slope and intercept of the straight lines. The pseudo-second-order rate constants are derived based on the sorption capacity of the solid phase, where K2 is the rate constant for the pseudo-second-order model. Determined experimentally by plotting t/q against t. The mean free vitality of adsorption (E) was figured as 2.62 kJ mol^−1^ and the extent of E communicated gives data on the adsorption mechanism. An E value ranging from 1 to 8 indicates physisorption and 8–16 kJ mol^−1^ predicts ion exchange. Thus, the evaluated value of 2.62 kJ mol^−1^ predicts the phenomenon of physisorption, which suggests that metal ions were favorably adsorbed by this biosorbent in a multi-layer fashion. The overall result suggested that 98.2% of U (VI) by biosorption of U in the mechanism of adsorption will include chemisorption mechanistic pathway: Langmuir, Freundlich, equations and the values of K_f_ 5.791 where K_L_ 3.9 were determined from the linear plot of log q_e_ vs. log C_e_ at 30 °C, indicating that metal ions were favorably adsorbed by this biosorbent in a multi-layer fashion and instrumentation of beads characterizing novel Binding sites using FTIR & SEM beside change in peaks position which assigned for its groups confirm biosorption of metal.

## Introduction

This isotherm is a blind equation and is widely used for the description of adsorption, including the adsorption of natural and inorganic mixes on a wide assortment of adsorbents. q_e_ is the amount adsorbed, K_f_ is the characteristic constant related to the adsorption capacity, C_e_ is the equilibrium concentration, and n is the specific consistency related to the level of ideality of adsorption. When 1/n = 1, the value of K_f_ is determined by the units in which q_e_ and ce are expressed^1^.

Kinetic models have been used to test the experimental data. In addition, information on the kinetics of metal uptake is required to select the optimum conditions for full-scale batch metal removal processes. The solute evacuation rate, which regulates the sorbate's live arrangement time at the solid arrangement interface, is how adsorption energy is conveyed^2–5^.

The pseudo-second-order kinetic parameters were determined using both linear and nonlinear techniques. Kinetic analysis is required to optimize various conditions for the biosorption process. Pseudo-first-order and pseudo-second-order models were used to study the kinetics of metal sorption. This kinetic model was developed based on the observation that the change in metal concentration with respect to time is proportional to a power of 1. A model for pseudo-second-order kinetics explains the sorption mechanism over the entire range of contact time in a pseudo-second-order kinetic model^6^. The model as written has the highest coefficient of determination; constants of the pseudo-second-order rate were determined experimentally by plotting t/q vs. t. This approach suggests that the rate-limiting step in the biosorption of heavy metals is chemisorption, which involves valence forces through the sharing or exchange of electrons between sorbent and sorbate (complexation), and is why the nonlinear method is thought to be a better way to determine the desired parameters, coordination, and/or chelation^2,3^. Several isotherm models, including the Freundlich and Langmuir models, are used to analyze the equilibrium behavior of single-component adsorption. According to the Freundlich model, adsorption occurs on a heterogeneous surface, which is a non-traditional approach. However, according to the Langmuir model, adsorption takes place at an adsorbent surface with homogeneous active sites. The pollutants interact and compete with one another as a result of the sewage's various components. Since single-component systems cannot be represented by the models used for multicomponent systems, they cannot be used. This is caused by various factors, including the complex mechanics involved in multicomponent adsorption. There has been extensive use of advanced models. As a result, the isotherm has just one component, while multi-component models are modified and explained below^6^.

## Methods

Heavy metal adsorption by immobilized bacteria immobilized by calcium alginate beads was prepared by dispensing a 3% (w/v) sodium alginate solution containing 3 mL of a 100% (v/v) *E. coli* cell-free extract. *E. coli S6 beads were dropped into a continuously stirred (250 rpm) 2% (w/v) CaCl*_*2*_* solution and left in the CaCl*_*2*_* solution overnight.* Bacterial cells were set up according to Leung et al.^7^ and were maintained in the conical flask containing 50 ml of samples for incubation, after which the specimens were pulled back for substantial metal examination by utilizing the titration method of uranium conc. The uranium content of the sample, the prepared standard solution, and the treated solution was measured at predetermined intervals in accordance with the method developed by the Nuclear Materials Authority and described by Davies and Gray in 1964. The results were utilized to fit various isothermal kinetic equations of adsorption, such as Langmuir, Freundlich, and isothermal models^2^, in batch mode with U (VI).

The determination of kinetic parameters of free and immobilized cells was performed for measuring reaction rates and kinetic constants at different metal concentrations, U (mg^−1^), q_e_ (mg^−1^), and q_e_ = 0.48 ((mg/g^−1^)) at the optimum reaction conditions. (K_m_ and V_max_) were calculated from the slope and intercept of the straight lines, respectively.

### Determination of uranium

The uranium content of the sample, prepared standard, and treated solution were determined according to the method described by and^8,9^.

#### Reagents

1. Orthophosphoric acid (H_3_PO_4_, 85%)
2. Conc. (HCl, 32%)
3. 10% ammonium ferrous sulphate(10 gm A.F. S + 10 ml H_2_so_4_ conc. then up to volume of 100 ml with distilled water).
4. Titanium trichloride (TiCl_3_)
5. Sodium nitrite (NaNO_2_)
6. 20% urea solution
7. Sodium salt (0.2 g diphenyl amino-4-sulfonic acid sodium salt + 0.2 sodium carbonate, then add drops of dist. H_2_O with stirring and up to volume 100).
8. Ammonium meta vanadate (NH_4_VO_3_).

#### Procedure

- In a dry and clean 100 ml Erlenmeyer flask, 5 ml of uranium sample was placed, and the following chemicals were added in the same order.
- ten ml of double-distilled water
- 10 ml (H_3_PO_4_)
- 1 mL conc. (HCl).
- 5 drops of 10% ammonium ferrous sulphate.
- 2–3 drops of Ticl_3_ were added till the solution changed to a purplish color.
- The reaction was left for 5 min.
- 3 drops of 15% NaNo_2_ were added till the brownish color appeared, then disappeared. Immediately, 5 ml of (urea 20%) was added and followed by rapid shaking till the air bubbles were stopped.
- The reaction was left again for 2 min before adding the indicator.
- 2 drops of indicator sodium salt were added.
- Titration against 0.1 Ammonium meta-vanadates was performed till the end point of pale violet color appeared.

The uranium concentration was calculated according to the following equation:

$${\text{U }}\left( {{\text{mg}}/{\text{l}}} \right) \, = {\text{ T }} \times {\text{ V1 }} \times { 1}0^{{3}} /{\text{v}}$$

where (T) is the titration intensity of NH_4_VO_3_ Solution, (V1) is the consumed volume of NH_4_VO_3_ Solution, and (v) solution is the volume of the measured sample.

### Materials

Ammonium meta-vanadates (NH_4_VO_3_) from biomaterials India, indicator sodium salt (0.2 gm diphenyl amino-4-sulfonic acid sodium salt + 0.2 g sodium carbonate from biomaterials India, titanium trichlorid (Ticl_3_) from biomaterials India, Hidrochloric acid (HCl) from biomaterials, orthophosphoric acid (H_3_PO_4_) from biomaterials, Sodium nitrite (NaNO_2_) from biomaterials, 20% Urea Solution co (NH_2_)_2_ from biomaterials, sodium alginate From sigma alderich , NaNo_2_ sodium Nitrite from biomaterials .

## Results and discussion

### Kinetic study of isotherm models

**Figure 1:**
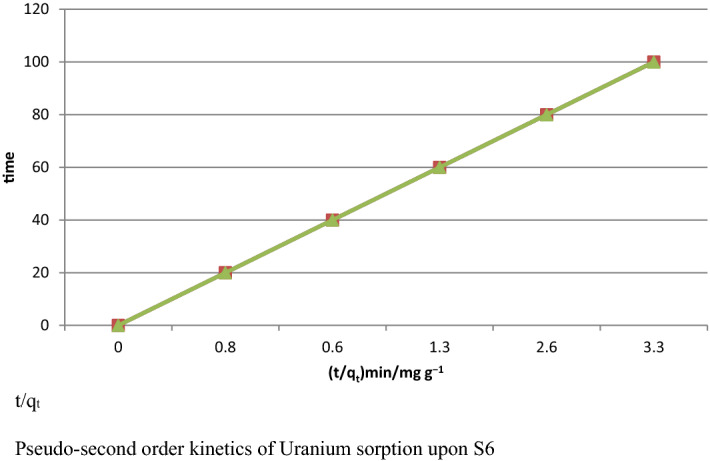
Pseudo-second order kinetics of Uranium sorption upon S6.

**Figure 2:**
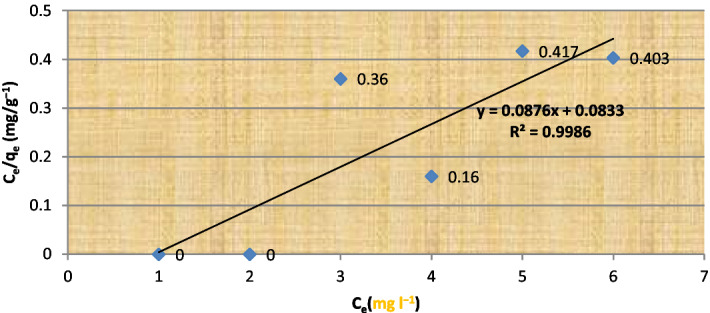
Langmiur isotherm of U (VI) ion adsorption on alginate beads.

**Figure 3:**
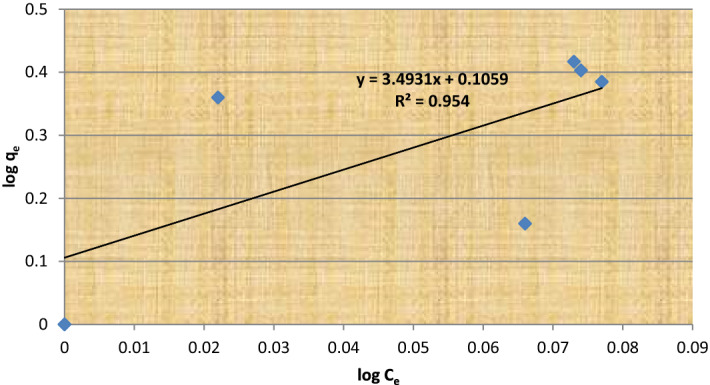
Linear plot of log q_e_ vs. log C_e_ as at 30 °C from the Freundlich isotherm of U (VI) adsorption on alginate-S6 *E. coli as biosorbent*.

The pseudo-second-order rate constants were tentatively resolved by plotting t/q against t, as shown in Figs. 1, 2, and 3. Compared to pseudo-first-arrange energy, the pseudo-second-arrange display as composed has the highest coefficient of assurance; this model is thought to be more appropriate to describe the kinetic data in biosorption systems^10^. Our findings were put to use to fit a number of isotherm kinetic equations for adsorption, including Langmuir, Freundlich, and isotherm models with U parallel with^2^ findings.

Bio-sorption and slow phase involved active metabolism-dependent transport of metal into the bacterial cells. Although there may have been some concurrent ion inhibition when the modified alginate beads were used to remediate 10 mg^−1^ U (VI), bioremediation ultimately prevailed, characterization of the newly synthesized product and proof of the physical nature of the newly synthesized alginate materials that are to be cited here in this article The idea of the coupling sites and their inclusion during biosorption can be roughly examined with the help of FTIR. As a result, we calculated the coupling points using FTIR instruments We used FT-IR spectra to confirm the availability of binding sites, as shown in Tables 4 and 5. Data of FT-IR OF Unloaded *E. coli*. (S6) Bacterial Isolation as indicated in tables after loading For uranium, we discovered amino acid (O–H) stretching, protein (N–H) stretching, phosphate C–O stretching band, P–H stretching, protein amide I band primarily (C=O) stretching, protein (CH_2_) and (CH_3_) bending of methyl, lipid (CH_2_) bending of methyl, carbohydrate (c-o) of polysaccharides, nucleic acid (other phosphate containing compound), >p=o stretching.

Whereas regarding our study under publication, "Application of the FTIR spectra of U-loaded and unloaded free and immobilized cells and Scanning Electron Microscopy (SEM) The microcapsule system had good mechanical strength, flexibility, and biocompatibility between the *E. coli* capsule and the microcapsule. In addition, the internal, three-dimensional network structure of the microcapsule provided sufficient spaces for *E. coli* capsule growth and good encapsulating stability. Scanning electron microscopy of these beads, the synthetic solution in the sample, and the control showed that they were hollow from the inside (having smooth inner walls). In SEM/EDS analysis of the Ca-alginate beads after the experiment, void spaces of the beads were found to be filled with precipitates of heavy metals, showing that Ca-alginate beads can be successfully used as a biosorbent for the removal of uranium, which agreed with^11–14^. Analysis revealed that the carboxyl and amino groups were responsible for metal binding Negatively charged and easily accessible carboxyl groups are essential for the binding of metal captions^12–15^. It has been observed that potentiometric titrations can be used to gather data on the types and numbers of binding sites. *Pseudomonas aeruginosa* was titrated by^15^, who also When *E. coli* was grown in the presence of U (VI), it was discovered that the unstained whole mount of *E. coli* interacted with the metal. Since the whole cells were collected, the contrast seen in the micrographs was due to the binding/accumulation of metallic U only^16^.

At various beginning metal concentrations, straight-line graphs of log (q_e_ − q_o_) against t were generated hypothetically to determine the rate constants and equilibrium metal uptake^17^. The experimental value is then contrasted with the q_e_ value obtained using this procedure. The heat of adsorption can be used to determine whether the biosorption process is exothermic or endothermic. The Langmuir constant, K_L_, and temperatures T, where K_o_ is the adsorption equilibrium constant, E in (kJ mol^−1^) is the activation energy of adsorption/heat of adsorption, R is the gas constant (0.0083 kJ/mol^−1^ K^−1^), and T is the absolute temperature (K)^18^.

The percentage of remediation of U (VI) was calculated as U (VI) % = (C_i_ − C_f_)/C_i_ * 100, where C_i_ = initial concentration and C_f_ = final concentration. The residual U (VI) in the medium was quantified by titration. At the typical remedial rate (R) of 100 ppm U (VI) m^−3^ of water per day, it was discovered. The final result was attained after five repetitions when there was no detectable U (VI) in the container. For initial concentrations of 100 of U (VI) (Tables 1 and 2), the values of q_e_, or the quantity of metal adsorbed (in mg/g) on the bead biomass, were determined using Eq. (1)

1 $${\text{C}}_{{\text{e}}} /{\text{q}}_{{\text{e}}} = {\text{ C}}_{{\text{e}}} /{\text{q}}_{{\text{m}}} + {1}/{\text{b}}*{\text{q}}_{{\text{e}}}$$

**Table 1:** Equilibrium adsorption capacity q_e_ (in mg/g^−1^) for various U concentration in ppm, C_o_ (mg l^−1^) q_e_ (mg/g^−1^), q_e_ = 0.048 (mg/g^−1^).

U conc. (ppm)	q_e_ (mg/g^−1^)
10	0.012
50	0.024
100	0.048
250	0.12
500	0.24
1000	0.5

**Table 2:** Equilibrium adsorption capacity q_e_ (in mg/g^−1^) for various U concentration in ppm, C_o_ (mg l^−1^) q_e_ (mg/g^−1^), q_e_ = 0.048 mg/g^−1^ for immobilized cells.

Immobilized cells	C_e_/q_e_	C_o_ (mg l^−1^)	Log C_e_ (mgl^−1^)	log q_e_ (mg/g^−1^)	q_e_ (mg/g^−1^)	Time (min)
0	0	0.00	00	1.3	0.048	0
78	0.360	0.022	1.6	1.2	0.061	20
34	0.160	0.066	1.1	0.3	0.41	40
27	0.417	0.073	1.1	0.75	0.175	60
26	0.403	0.074	1.13	0.735	0.184	80
23	0.385	0.077	1.14	0.735	0.20	100

The results were fitted with isotherm models of Langmuir and Freundlich. A plot of C_e_/q_e_ vs. C_e_ (Fig. 2).

According to Eq. (1), the square of the regression coefficient, R2, was calculated to be 0.9986, indicating that the Langmuir isotherm could not be the perfect model but completely describe the adsorption, Where from Freundlich isotherm Eq. (2)

2 $${\text{Log }}\left( {{\text{q}}_{{\text{e}}} } \right) \, = {\text{log }}\left( {{\text{K}}_{{\text{f}}} } \right) \, + {1}/{\text{n log }}\left( {{\text{C}}_{{\text{e}}} } \right)$$

the linear plot of log q_e_ vs. log C_e_ as at 30 °C (Fig. 2), and the values of K_f_ (5.791), which revealed that metal ions were positively adsorbed by this biosorbent in a multi-layer form. The adsorption data were also plotted as RT l_n_ [1 + 1/C_e_] vs. l_n_ q_e_.

Adsorption capacity is the most important characteristic of an adsorbent. It is defined as the volume of adsorbate that the adsorbent can hold in one unit of mass. As the interaction between sorbent and solute molecules is anticipated to be strong, various mechanisms may be at play. Several characteristics, including specific surface area, cation exchange capacity, and specific volume, affect this value^19,20^. For example, hydroxyl, carbonyl, carboxyl, sulfhydryl, thioether, sulfonate, amine, imine, amide, imidazole, phosphonate, and phosphodiester groups may be present within the biosorbent structure, and adsorption will not be limited to physical bonding^21–23^.

All of this data points to the functional groups listed in Tables 5 and 6 as being in charge of the uptake of metals in our bacterial biomass. Also supporting the biosorption of metal ions from waste due to ion charge interactions is the change in peak locations ascribed to its groups. When Tables 3, 4, and 5 were compared, we discovered a rise in the number of binding sites, which shows that immobilized bacteria have high efficiency for metal uptake and also alters the peak positions assigned to its groups, confirming the biosorption of metal ions from waste due to ion charge interactions (Table 6).

**Table 3:** Langmuir isotherm models constants.

Isolate no.	Practical capacity q_e_ mg/g	Q_max_	K_L_	R^2^
S6 *E. coli*	77	98.2	3.9	0.9986

**Table 4:** Freundlich isotherm models constants.

Isolate no.	Practical capacity q_e_ (mg/g)	Q _max_	K_f_	R^2^
S6 *E. coli*	77	89	5.791	**0.954**

Significant values are in bold.

**Table 5:** Shows the FT-IR data of an unloaded immobilized *E. coli* (S6) bacterial isolate.

Main peak (cm^−1^)	Intensity of loaded band	Typical band	Wavenumber range
1-3436.42	71.14	Amino acid (O–H) stretching protein v (N–H) stretching	3029–3639
2-1725.23	97	Phosphate C–O stretching band, P–H stretching	2344–2365
3-1636.3	92	Protein amide I band mainly (C=O) streching	1583–1709
4-1380.99	86	Protien (CH_2_) and (CH_3_) bending of methyle lipid (CH_2_) bending of methyl	1425–1477
5-1354.15	80	Carbohydrate (c-o) of polysaccharides, Nucleic acid (other phosphate containing compound) >p=o stretching of phosphodiester	1072–1356
6-1038.48	91.90	Carbohydrate (c-o) of polysaccharides, Nucleic acid (other phosphate containing compound) >p=o stretching of phosphodiester	1072–1356
7-924.7.363	99	Acid chlorides CCl stretch	730–550
8-879.577	99	Acid chlorides CCl stretch	730–550
9-808.992	98	Acid chlorides CCl stretch	730–550
10-597	88	Acid chlorides CCl stretch	730–550

**Table 6:** Shows the FT-IR data of an unloaded immobilized *E. coli* (S6) bacterial isolate.

Main peak (cm^−1^)	Intensity of loaded band	Typical band	Wavenumber range
1-3909.42	96.64	Amino acid (O–H) stretching protein v(NH) stretching	3029–3639
2-3859.23	96	Phosphate C–O stretching band, P–H stretching	2344–2365
3-1636.3	92	Protein amide I band mainly (C=O) streching	1583–1709
4-1729.99	93	Protien (CH_2_) and (CH_3_) bending of methyle Lipid (CH_2_) bending of methyl	1425–1477
5-1630.15	85	Carbohydrate (c-o) of polysaccharides, nucleic acid (other phosphate containing compound) >p=o stretching of phosphodiester	1072–1356
6-1429.4	90.90	Carbohydrate (c-o) of polysaccharides, nucleic acid (other phosphate containing compound) >p=o stretching of phosphodiester	1072–1356
7-1382.7	80	Acid chlorides C–Cl stretch	730–550
8-1175.577	100	Acid chlorides C–Cl stretch	730–550
9-1037.5.992	90	Acid chlorides C–Cl stretch	730–550
10-936.27	103	Acid chlorides C–Cl stretch	730–550
11-880.34	104.166	Acid chlorides C–Cl stretch	730–550
12-818.634	101	Acid chlorides C–Cl stretch	730–550
13-664.357	95	Acid chlorides C–Cl stretch	730–550
14-562.148	92	Acid chlorides C–Cl stretch	730–550

### Energy dispersive X-ray (EDX) and scanning electron microscopy.

**Figure 4:**
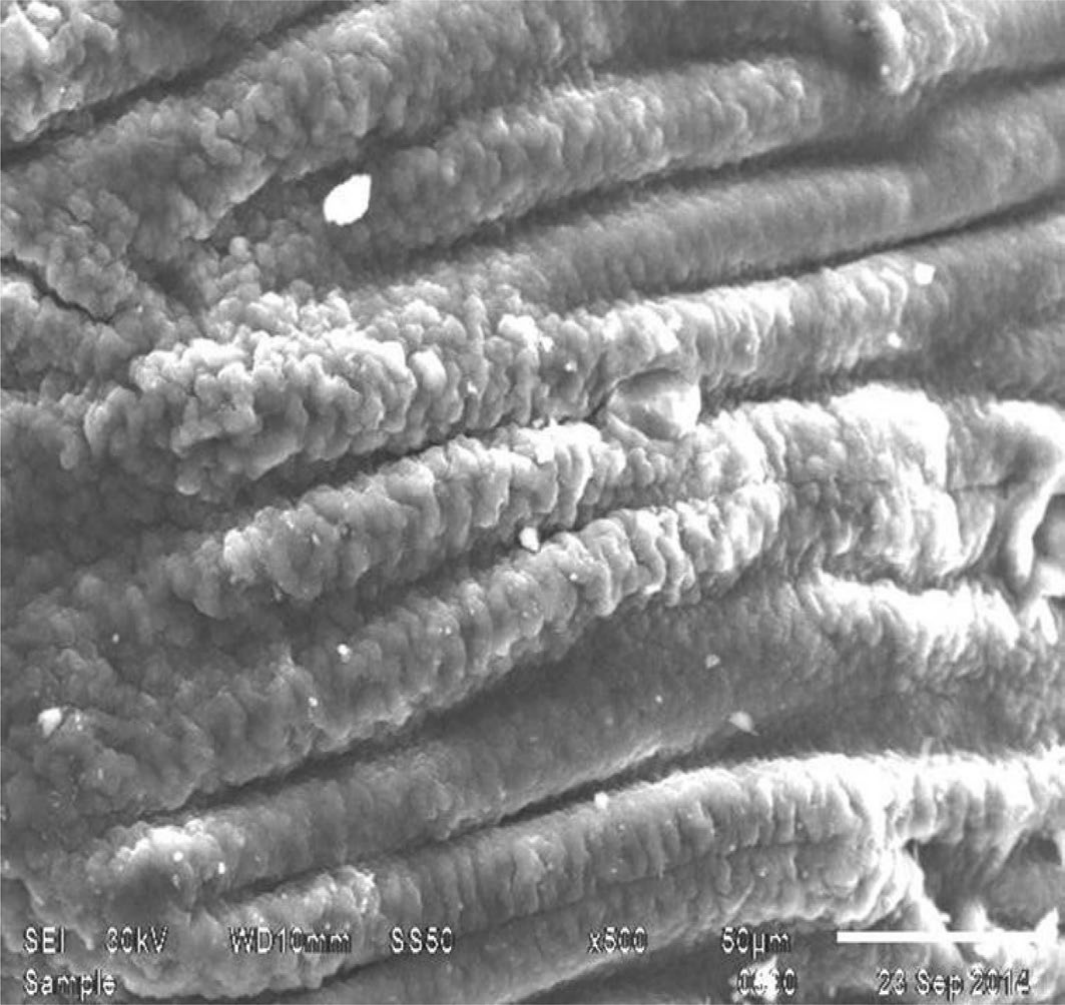
(EDX) to confirm the biosorption of U by immobilized cell-free extract-loaded Ca-alginate beads.

**Figure 5:**
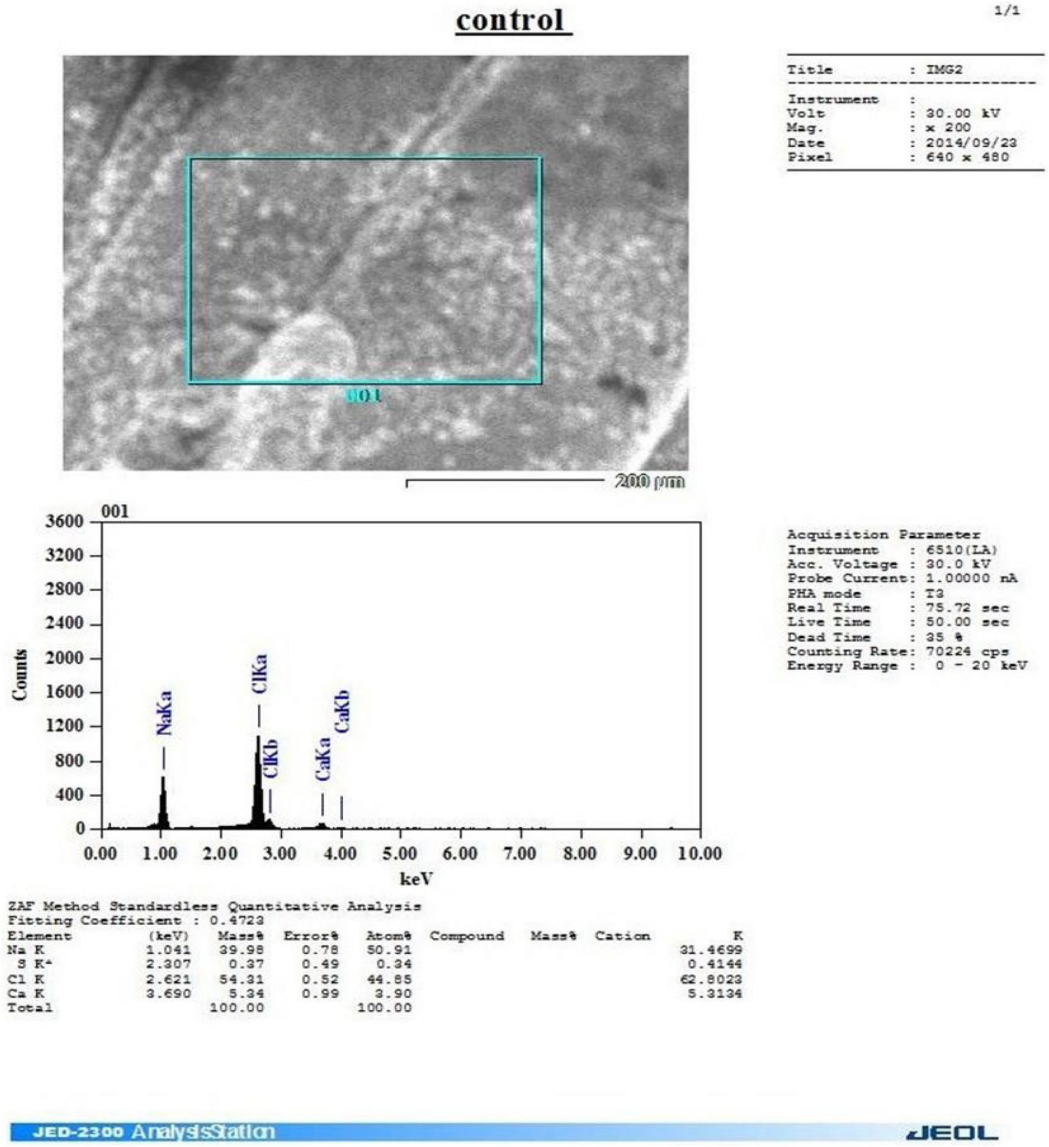
Cell-free extract loaded Ca-alginate beads.

**Figure 6:**
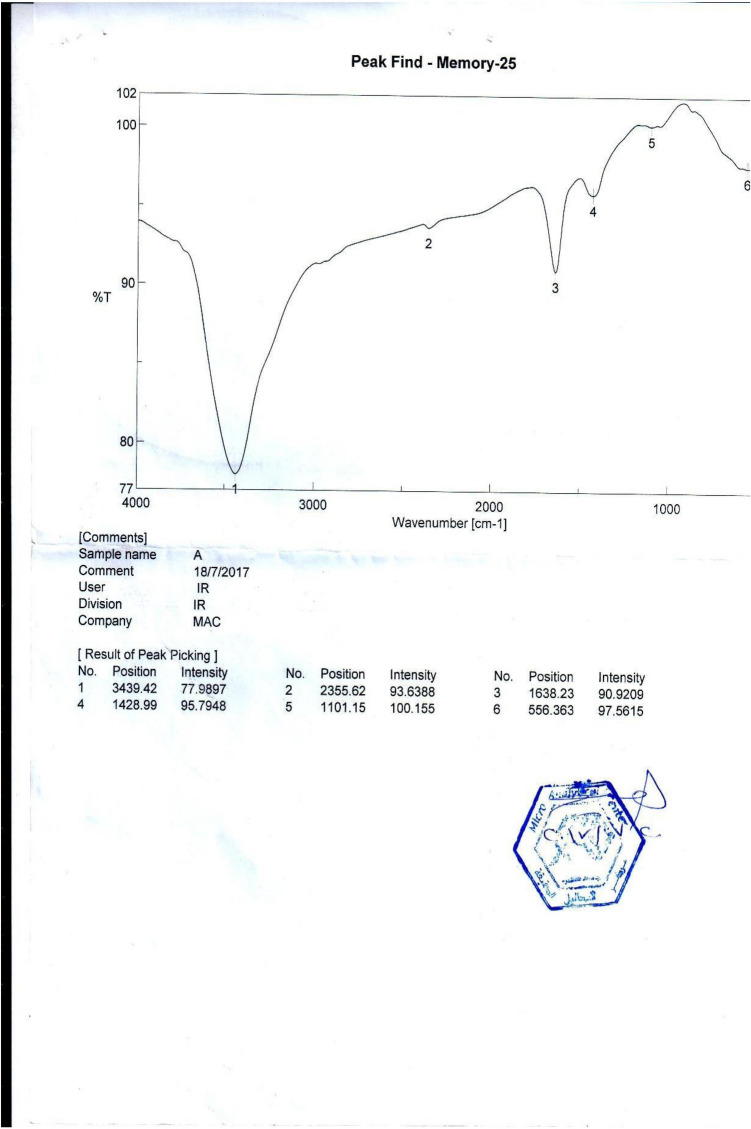
IR spectrum of live *E. coli* before the biosorption process.

Alginate beads (Figs. 4 and 5), predominantly ellipsoidal spheres, with an average diameter of 3–5 mm were used in the packed bed to remediate 10–1000 ppm U (VI) in a synthetic uranium solution. The effectiveness of different dosages of beads was considered, and the optimized ratio of 1:5 (v/v) of beads to water was used in all batch studies of isotherm kinetics. Scanning electron microscopy of these beads, synthetic solution (Figs. 3 and 4), and control (Fig. 2), showed that they were hollow from inside (having smooth inner walls). In SEM/EDS analysis of the Ca-alginate beads after the experiment, void spaces of the beads were found to be filled with precipitates of heavy metals, showing that Ca-alginate beads can be successfully used as a biosorbent for the removal of uranium, which agreed with^24^ as in Figures 4, 5, 6, and 4, as seen in Figs. 4 and 5. Uranium biosorption has been confirmed in the spot zone.

### Isotherm parameters value

It has been stated that the extent of E suggests an adsorption mechanism. E values ranging from 1 to 8 indicate physiosorption and 8–16 kJ mol^−1^ predict ion-exchange. The mean free vitality of adsorption (E) was stated as 2.62 kJ mol^−1^^2,25,26^. According to the overall findings, 98.2% of U (VI) was absorbed through the biosorption of the U models used to compute adsorption capacity: Equations show that the same beads might be utilized for biosorption for 15 days, thus the evaluated value of 2.62 kJ mol^−1^ This suggests physiosorption as the process but also mentions chemisorption, which involves valence forces through the sharing or exchange of electrons between the sorbent and the sorbate, complexation, coordination, and/or chelation^2,3,27^. The heat of the adsorption of metal ions suggests that a physical mechanism underlies adsorption. It was thought that the diffusion of heavy-metal ions into the porosity of beads was the rate-determining step of the adsorption since the diffusion profile of the bead technique was similar to the profile of active carbon, which physically adsorbs the heavy metal ions^23,28,29^. According to the researcher cited below, because the pores of the beads were clogged with deposited copper, the researcher here shows that the porous structure of the host polymer has a large number of pores. In the outer part of the beads, clogging or blocking of the pores may occur due to the Cuo deposit, it is probable that the metal ions were adsorbed during the formation of a multi-layer. Therefore, forces of physical attraction might have been at fault. Therefore, the adsorption may have resulted from physical attraction forces. The dense pore structure and rough surface of kaolin can offer the physical space required for the chemical adsorption of heavy metals at high temperatures. A certain surface area, pore size, and pore volume offer a richer physical adsorption space and more active sites for the chemisorption of heavy metals^30–34^ (Fig. 6).

The endpoint was reached after 5 times when there was no amount of U (VI) measured in the present container. The values of q_e_, or the amount of metal adsorbed (in mg/g) on the bead biomass, were calculated using Eq. U (VI) % = (C_i_ − C_f_)/Ci * 100, where C_i_ = initial concentration and C_f_ = final concentration as the interaction between sorbent and solute molecules is expected to be strong, various mechanisms may be involved. Adsorption capacity is the most important characteristic of an adsorbent^19,20,35^. R is a universal gas constant, and T is the absolute temperature (K). The heat of adsorption can be calculated from the plots of log b versus 1/T 19, 24^36–38^. According to^39–41^ Tables 2, 3, and 4 provide the parameters and the typical percent % biosorption practically and expected theoretically as shown the in tables. The equilibrium isotherm was of a favourable kind, where the Langmuir equation accurately captures the results of our experimental work. The monolayer coverage of the metal ions on the surface of the biomass is shown by the application of the Langmuir isotherm. The overall result suggested that 98.2% of U (VI) by biosorption of U in the mechanism of adsorption will include chemisorption mechanistic pathway^42–44^.

## Conclusion

The kinetic constants for free and immobilized cells were determined by measuring reaction rates at different metal concentrations, U (mg^−1^), q_e_ (mg g^−1^), and q_e_ = 0.48 mg g^−1^ at the optimum reaction conditions. The average free vitality of adsorption (E) was calculated to be 2.62 kJ mol^−1^. The extent of E communication gives data on the adsorption mechanism. An E value ranging from 1 to 8 indicates that the adsorption mechanism is physiosorption and a range from 8 to 16 kJ mol^−1^ predicts indicates that the adsorption mechanism is ion-exchange. Thus, the evaluated value of 2.62 kJ mol^−1^ and this predicts the adsorption mechanism as physiosorption but also includes chemisorption. The overall result suggests that 98.20% of U (VI) by biosorption of U in the mechanism of adsorption will not be restricted to physical bonding, and the kinetic model of pseudo-second-order represents the experimental data, accurately, the Langmuir model of favorable type for the biosorption isotherm indicating that metal ions were favourably adsorbed by this biosorbent in a multi-layer fashion, With characterization of novel binding sites using FTIR and SEM, as well as a change in peak position assigned to its groups.

## Data Availability

All data generated or analyzed during this study are included in this published article.

## Abbreviations

C_i_: Initial concentration
C_f_: Final concentration
q: Adsorbative capacity per unit mass of adsorbent [mmol/g]
K_L_: Langmuir constant
K_f_: Freundlich constant
q_t_: Adsorbed amount on the biosorbent at time t [mg/g]
R^2^: Correlation coefficient
R: Universal gas constant [cal/mol K]
T: Absolute temperature [K]
